# Association of age and insulin resistance with sex hormone-binding globulin levels in healthy men

**DOI:** 10.20945/2359-4292-2024-0360

**Published:** 2025-08-25

**Authors:** Indianara Franciele Porgere, Bruna Martins Rocha, Gustavo Monteiro Escott, Luiza Carolina Fagundes Silva, Priscila Aparecida Correa Freitas, Fabíola Satler, Sandra Pinho Silveiro

**Affiliations:** 1 Programa de Pós-Graduação em Ciências Médicas: Endocrinologia, Universidade Federal do Rio Grande do Sul, Porto Alegre, RS, Brazil; 2 Serviço de Patologia Clínica, Hospital de Clínicas de Porto Alegre, Porto Alegre, RS, Brazil; 3 Serviço de Endocrinologia, Hospital de Clínicas de Porto Alegre, Porto Alegre, RS, Brazil

**Keywords:** Testosterone, Sex hormone-binding globulin, Insulin resistance

## Abstract

**Objective:**

To evaluate the putative association of age and insulin resistance with sex hormone-binding globulin levels in healthy men.

**Methods:**

In total, 136 healthy men without obesity, aged 18 years or older, were included. Total testosterone was measured by electrochemiluminescence, and sex hormone-binding globulin by chemiluminescence. Calculated free testosterone was obtained by Vermeulen's equation. Insulin resistance index was estimated as triglycerides/HDL ratio.

**Results:**

The sample was divided into tertiles according to age (18 to 29; 30 to 49; 50 to 67 years). Sex hormone-binding globulin levels were higher in men > 50 years old compared to those of the second and first tertiles (41 ± 17 *versus* 35 ± 12 and 29 ± 9 nmol/L; p < 0.001), while values of calculated free testosterone were lower in the older tertile (7.7 ± 1.9 *versus* 8.8 ± 2.2 and 10.4 ±3.1 ng/dL; p < 0.001). Age did not influence total testosterone levels. Insulin resistance index was inversely and significantly correlated with sex hormone-binding globulin (r = -0.371; p < 0.001).

**Conclusion:**

There is a significant increase in serum sex hormone-binding globulin in older healthy men, highlighting the need for age-specific reference values. Furthermore, insulin resistance seems to reduce this globulin levels, perhaps pointing out low sex hormone-binding globulin as a putative predictor of related chronic diseases

## INTRODUCTION

Sex hormone binding globulin (SHBG) is a protein that plays a central role in regulating the transport, bioavailability and metabolism of sex steroid hormones such as testosterone (T) (^1^). In men, 40% of total testosterone (TT) is strongly bound to SHBG, 58% is weakly bound to albumin (bioavailable T), and the remaining 0.5 to 2% circulates in free form, namely as free T (FT) (^2^).

Produced by the liver, SHBG serum levels are regulated by sex hormones as well as by several other hormones and non-hormonal factors. Consequently, SHBG levels might vary under either physiological or pathological conditions, such as pubertal and senescence changes (increase) or in metabolic syndrome (decrease) (^3^). Measurement of SHBG in clinical practice has been evaluated as a potential disease biomarker to assess risk for type 2 diabetes mellitus (DM), cardiovascular events, and liver disease (^3^).

The literature suggests that SHBG levels increase upon aging (^4^). For instance, the European Male Aging Study showed an age trend for SHBG of increasing 0.65 nmol/L per year (^5^). On the other hand, an age-related gradual decline (approximately 0.5% per year) in serum T has been described (^6^), with a more pronounced decline of FT than TT due to the confounding increases of SHBG (^7^). Therefore, these changes have raised questions regarding the role of sex hormone levels in male aging and indicating the need of age-related reference values for TT, FT, and SHBG in clinical practice.

TT measurement is currently the standard approach to evaluate androgen levels (^8^). However, TT may not be a reliable measure in the presence of conditions that affect SHBG levels, when the fractions of FT and BT (bioavailable T) should be used instead to evaluate androgen levels (^2^,^4^).

This study aimed to evaluate the putative association of age and insulin resistance with SHBG levels in healthy men.

## METHODS

### Participants

A cross-sectional study was conducted in a sample of healthy men without obesity. Participants were blood donors recruited from the blood bank of the university hospital, *Hospital de Clínicas de Porto Alegre* (HCPA), from November 2017 to November 2019. Participants from the biobanking of HCPA were also included (^9^). Blood samples were taken in the morning after a standard breakfast (calculated as 50 g carbohydrate, 14 g fat and 11 g protein), as this is the routine recommendation before blood donation procedures.

Eligibility criteria required participants to be aged ≥18 years and to present a body mass index (BMI) < 30 kg/m^2^, and no known disease. Individuals with DM, hypertension, or cancer diagnosed in the prior 5 years were excluded.

Participants self-reported data as skin-color, medical conditions, smoking, alcohol intake, prescription drugs, and symptoms of hypogonadism. As recommended by the endocrine society guidelines, symptoms suggestive of hypogonadism, such as reduced sexual desire and erectile dysfunction, were investigated. (^10^). Blood pressure was measured using a validated Omron device (Model HEM-7200), as recommended by the guidelines (^11^). Participants weight (kg) and height (m) were evaluated to calculate BMI (kg/m^2^).

### Laboratory methods

Serum TT was measured using an electrochemiluminescence immunoassay (Abbott GmbH & Co. KG), and serum SHBG was assessed by an chemiluminescence assay (Abbott GmbH & Co. KG). Between- and within-assay coefficients of variation for TT were 5.72% and 5.43%, and for SHBG 3.87% and 3.84%, respectively. Calculated free testosterone (cFT) was estimated based on TT, SHBG, and albumin, using the Vermeulen's equation (^12^).

Glycated hemoglobin (HbA1c) was quantified by an ion-exchange high performance liquid chromatography (HPLC) (Merck-Hitachi L9100 Analyser, Merck, Dermstadt, Germany), National Glycohemoglobin Standardization Program (NGSP) certified. Serum albumin was measured by colorimetric method, with a 2.01% coefficient of variation. Total cholesterol and high-density lipoprotein cholesterol (HDL-c) were measured by enzymatic methods. Blood count was assessed by light absorbance/impedance/flow cytometry method. C-reactive protein (CRP) was measured by turbidimetry immunoassay. Thyrotrophin (TSH) was measured by chemiluminescence of microparticles immunoassay method. Serum creatinine was measured by a traceable Jaffe kinetic method. Glomerular filtration rate (GFR) was estimated using the CKD-EPI equation (^13^).

The presence of insulin resistance was verified by the insulin resistance index (IRI): triglyceride/high-den-sity lipoprotein cholesterol ratio (TG/HDL-c) (^14^,^15^).

### Ethics statement

The study was approved by the Research Ethics Committee of HCPA (project number 20190732), and the study participants provided written informed consent in accordance with the Declaration of Helsinki.

### Statistical analysis

Results were expressed as mean ± standard deviation (SD), percentages, median (minimum, maximum), or percentiles.

To investigate the association between SHBG, TT, and cFT with age, the participants were stratified in tertiles of age. Variables with normal distribution were evaluated by one-way analysis of variance (Anova), followed by Tukey's post hoc test. Variables without normal distribution were evaluated using Kruskal-Wallis and Dunn's post hoc test. Pearson's and Spearman's correlation coefficients were also used.

Multiple regression analyses were used to simultaneously assess variables as predictors of TT, SHBG, and cFT levels.

To establish ranges for SHBG measurements in this population, the 2.5th percentile (p = 2.5) and the 97.5th percentile (p = 97.5) of the study sample were estimated, as recommended (^16^).

Sample size was estimated using the PSS Health online tool (^17^). A sample size of 25 participants was estimated in each age group to estimate SHBG values changes by age, with an absolute margin of error of 6.4 nmol/L and a 95% confidence level, considering 15.2 nmol/L as the expected SD, according to Krakowsky and cols. (^18^).

All analyses were carried out using SPSS software program version 18.0 (Statistical Package for the Social Sciences; SPSS Inc., Chicago, IL, USA) and R version 3.6.2 for macOS (R Foundation for Statistical Computing). For all analyses, a 2-tailed p < 0.05 was considered to indicate statistical significance.

## RESULTS

Our study included 136 healthy men, aged 41 ± 13 years (18 to 67 years), 79% self-declared White and 9% smokers. Participants reported no symptoms suggestive of hypogonadism. Table 1 summarizes the clinical and laboratory characteristics of the participants divided into age tertiles.

**Table 1:** Clinical and laboratory characteristics of healthy volunteers by age tertile

	Total sample (n = 136)	18 to 29 years (n = 46)	30 to 49 years (n = 47)	50 to 67 years (n = 43)	p-value
Age, years	41 ± 13	26 ± 4	42 ± 5	56 ± 5	By design
BMI, kg/m^2^	25 ± 3	24 ± 3	26 ± 2	25 ± 2	0.004*
SBP, mmHg	120 ± 10	122 ± 10	119 ± 9	119 ± 10	0.357
DBP, mmHg	73 ± 8	74 ± 9	72 ± 7	72 ± 8	0.617
HbA1c, %	5.1 ± 0.3	5.0 ± 0.2	5.1 ± 0.3	5.2 ± 0.3	0.222
Albumin, g/dL	4.8 ± 0.5	4.9 ± 0.4	4.8 ± 0.4	4.7 ± 0,6	0.405
GFR, CKD-EPI, mL/min/1.73m^2^	97 ± 18	109 ± 19	97 ± 13	85 ± 15	<0.001†
Total cholesterol, mg/dL	189 ± 40	170 ± 30	193 ± 40	205 ± 40	<0.001‡
HDL-c, mg/dL	51 ± 12	50 ± 10	50 ± 14	53 ± 12	0.540
Triglycerides, mg/dL	116 (47-390)	94 (47-384)	126 (54-356)	127 (56-390)	0.004^§^
IRI	2.1 (0.9-11.8)	1.9 (0.9-8.9)	2.6 (1.0-9.8)	2.6 (0.8-11.8)	0.064

* Statistical difference between the first and the second tertile; ^†^ statistical difference among all tertiles; ^‡^ statistical difference between the first tertile and the second and the last tertile; ^§^ statistical difference between first and last tertile.

Data presented as mean ± standard deviation or median (interval interquartile).

BMI: body mass index; SBP: systolic blood pressure; DBP: diastolic blood pressure; HbA1c: glycated hemoglobin; GFR: glomerular filtration rate; CKD-EPI: Chronic Kidney Disease Epidemiology Collaboration; HDL-c: high-density lipoprotein cholesterol; IRI: insulin resistance index.

**Figure 1:**
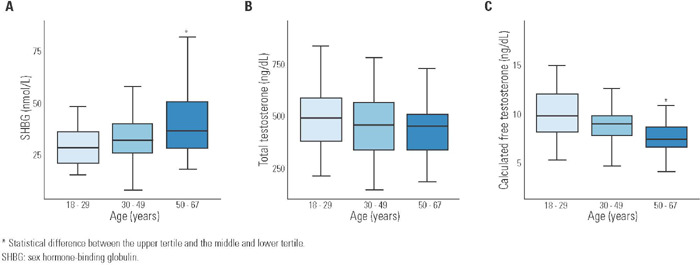
Boxplot of sex hormone-binding globulin in panel **A,** total testosterone in panel **B** and free testosterone in panel **C,** according to age tertiles.

By dividing our sample into tertiles of age, we found higher SHBG values in the upper tertile when compared to the intermediate and lower tertiles of age (41 ± 17 *versus* 35 ± 12 *versus* 29 ± 9 nmol/L; p < 0.001). cFT was significantly lower in the upper tertile compared to the middle and lower tertiles (7.7 ± 1.9 *versus* 8.8 ± 2.2 *versus* 10.4 ± 3.1 ng/dL; p < 0.001 (Figure 1). On the other hand, TT was similar among the three groups, and the values for the total sample ranged from 206 to 805 ng/dL

* Statistical difference between the upper tertile and the middle and lower tertile.

SHBG: sex hormone-binding globulin.

**Figure 2:**
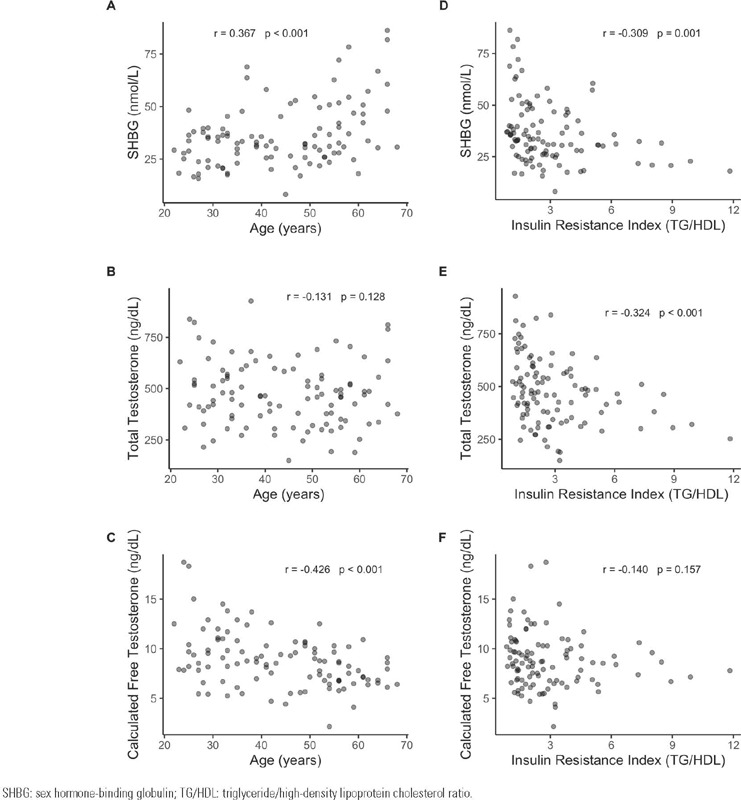
Correlations of sex-hormone binding globulin **(panel A and D),** total testosterone **(panel B and E)** and calculated free testosterone **(panel C and F)** with age and insulin resistance index.

A positive correlation was found between age and SHBG, and an inverse and significant correlation of age with cFT, but not with TT. When correlating IRI with SHBG and T fractions, we found an inverse and significant correlation with SHBG and TT, but not with cFT (Figure 2).

SHBG: sex hormone-binding globulin; TG/HDL: triglyceride/high-density lipoprotein cholesterol ratio.

We conducted a multiple linear regression to evaluate the association of age and IRI with the dependent variables SHBG, TT, and cFT. SHBG increased by 0.6 nmol/L per year of age, while cFT decreased by 0.08 ng/dL per year of age. No association was found between age and TT levels. SHBG and TT decreased with each increase in the IRI; 2.9 nmol/L (p < 0.001) and 0.07 (p < 0.001) ng/dL, respectively. However, we found no association with cFT.

Since the upper age tertile (≥ 50 years) had significantly higher SHBG values than the lower two tertiles, we analyzed the values of the 2.5^th^ and 97.5^th^ percentiles of the sample according to ages ≥ 50 years *versus* < 50 years to establish age-related values (Table 2). Considering that TT was similar among the three groups, the values represent the total sample.

**Table 2:** Values of sex hormone-binding globulin, calculated free testosterone, and total testosterone for men according to each age group

	SHBG (nmol/L)		cFT (ng/dL)		TT (ng/dL)	
Percentile (years)	2.5	97.5	2.5	97.5	2.5	97.5
18-49 (n = 93)	16	57	5	17	-	-
50-67 (n = 43)	20	82	4	11	-	-
18-67 (n = 136)	-	-	-	-	206	805

Considering that total testosterone was similar between groups, the values represent the total sample.

SHBG: Sex Hormone-Binding Globulin; cFT: calculated free testosterone; TT: total testosterone.

## DISCUSSION

This study shows that aging is followed by an increase in SHBG levels, and a reduction in cFT in healthy men without obesity, without significant variation in TT. Moreover, insulin resistance, as evaluated by IRI, was significantly associated with decreased serum SHBG and TT.

The positive association of serum SHBG with age has already been described in a study of over 150,000 men aged 40 to 69 years from the British Biobank (^19^). Other studies have also suggested the need for age-specific determination of SHBG, as SHBG levels tend to increase with age while TT levels tend to remain stable, and have also pointed to the central role of SHBG in men's health status (^20^,^21^). In line with these previous findings, our results suggest the adoption of an age-specific range for SHBG: from 16.0 to 57.0 nmol/L for men aged < 50 years and 20.0 to 82.0 nmol/L for men ≥ 50 years. Similar values were described in a study of 1,000 men from a medical center in the USA, in which younger men aged < 55 years also had lower SHBG (from 6 to 88 nmol/L) than older men (> 55 years, between 11 and 109 nmol/L) (^17^). A recent review (^22^) emphasized that the age-specific thresholds for SHBG proposed in the literature can also be observed in populations with chronic metabolic disorders. Wang and cols. (^23^) determined the SHBG reference range in a healthy sub-cohort of US adults who participated in the US National Health and Nutrition Examination Survey (NHANES) from 2013 to 2016. The study defined criteria for low SHBG as levels < 12.3 nmol/L in men < 50 years and < 23.5 nmol/L in men ≥ 50 years. Risk factors for low SHBG included higher BMI, diabetes, ethnicity (other than Hispanic, non-Hispanic Black, or non-Hispanic White), chronic obstructive pulmonary disease, coronary heart disease, and smoking. Approximately 66% of the study population was non-Hispanic White. The participants in our study also predominantly identified themselves as White (79%). In Brazil, as in many other countries, there is a mixture of several different origins (Black, White, Indigenous etc.). It is well known that self-reported ethnicity is not a reliable predictor of genomic ancestry (^24^,^25^). Therefore, we believe that a single domain, regardless of skin color, is more representative of the general population, as the values are consistent with those reported in other studies, including those involving more diverse groups (^18^,^23^). However, we recognize that future studies with more diverse populations are needed to further validate global applicability or, conversely, to establish ranges for individual populations.

Regarding cFT, we also found a marked decline in their levels in men aged > 50 years, in line with the literature (^4^,^26^). This is an expected phenomenon, since the increase in the carrier protein SHBG reduces the free fractions of T, while it mitigates the reduction of TT (^27^). Although this association has been extensively described, the reasons for the SHBG increase with aging are not fully understood yet. *In vitro* data on human hepatoma-cultured cells indicated that insulin-like growth factor 1 (IGF-1) may be a negative regulator of SHBG synthesis (^28^). Upon aging, IGF-1 decreases, reducing the inhibitory effect on SHBG production in hepatocytes, which increases SHBG levels (^29^). A large prospective cohort of 200,000 men suggested that higher free T and circulating IGF-1 are associated with an elevated risk of prostate cancer, whereas higher SHBG was associated with a lower risk (hazard ratio of 0.95 per 10 nmol/L of increment) (^30^). There are possible, nevertheless, confounding factors, since lower SHBG and higher IGF-1 levels are found in the presence of obesity (^4^,^31^), and it has been suggested that men with obesity are at increased risk of prostate cancer progression and high-grade forms of the tumor (^32^). Another possible explanation for the aging-related increase in SHBG is the independent increase of adiponectin with age (^33^), since this anti-inflammatory cytokine stimulates SHBG synthesis (^34^). Therefore, even though all this knowledge is not new, unfortunately, several laboratories do not take this phenomenon into account when reporting male hormone levels, including university hospitals.

Considering that SHBG levels interfere with aging-associated medical conditions, and metabolic profile understanding how age influences SHBG is essential, since it may have important clinical applications (^3^). After evaluating the association between SHBG and the risk of coronary heart disease (CHD) incidence in the United Kingdom Biobank (UKB), Li and cols. (^35^) demonstrated that elevated levels of SHBG were both directly and indirectly predictive of a lower risk of CHD in men and women. The relation between low SHBG concentrations and insulin-resistant states, such as type 2 diabetes and metabolic syndrome, could also be involved (^36^). In our study, we confirmed an inverse correlation between SHBG and insulin resistance, reinforcing that SHBG is independently associated with the risk of metabolic syndrome (^37^). Low SHBG has been suggested as a strong predictor of type 2 diabetes risk, with an odds ratio of 0.10 for men in the highest quartile of SHBG levels versus the lowest quartile (^38^). A decrease in TT levels has been described in hyperinsulinemia and obesity, related to lower SHBG levels (resulting from decreased liver production) or to a real decline in T production (^39^). Our study showed that there is a moderate and significant inverse correlation of the IRI with SHBG and TT. These results corroborate previous findings of a cohort of men with obesity, in which insulin resistance interfered in SHBG production and T levels (^40^). Considering that our study was conducted in men < 30 kg/m^2^, the correlations between IRI and SHBG may be present even in the absence of established obesity. In the UK Biobank study, smoking and alcohol intake were also found to reduce SHBG levels on top of a higher BMI (^5^). Therefore, well established positive lifestyle modifications, such as exercising on a regular basis and adopting healthy eating behaviors, might favorably affect age-related hormone homeostasis, improving the prognosis of related chronic conditions (^41^). SHBG has emerged therefore as a possible disease risk marker.

Unfortunately, for economic reasons, we were not able to determine the classical parameters of insulin resistance. Although we have used a surrogate marker of insulin resistance determined by TG/HDL-c, we believe that this less costly index is a useful and reliable indicator of cardiovascular risk. In a recent study of 802 consecutive patients undergoing coronary angiography for suspected CHD, the TG/HDL-c ratio and the TG/glucose index (TyG) were valuable predictors of the presence and severity of CHD (^42^). In another notable study of 403.335 UK Biobank participants who were free of cardiovascular disease (CVD) at baseline and followed for 8.1 years, 19.754 (4.9%) individuals developed CVD. There were significant trends toward increasing CVD risk across all quartiles of the TyG and TG/HDL-c ratio (^43^).

It is pivotal to have standardized and precise hormone dosages to assess, monitor, and intervene on serum levels of sex hormones and binding proteins (^27^). Furthermore, there is an urgent need to define sex steroid reference values and identify possible confounding factors that could influence these measurements. Therefore, it is highly recommended to establish values for each specific population, as advised by international guidelines (^44^). Besides proposing age-specific values for SHBG, in this study we propose a fixed range of 206 to 805 ng/dL as the value for TT, with no age-specific value, since we found no differences in values among age subgroups. Similar reference values 245 to 801 ng/dL for healthy men aged 18 to 74 years was proposed by Mezzullo and cols. (^27^), who also found no impact of age in TT levels. Moreover, in a healthy population without obesity of American and European men, aged from 19 to 39 years, the established reference range for TT was 264 to 916 ng/dL (^16^).

As the test subjects were voluntary blood donors, they were not fasting at the time of the morning blood sample. However, a nutritionist assessed their breakfast intake via interview and calculated a profile of about 40 to 50 g carbohydrate and 14 g fat intake.

Some studies suggest that an oral glucose tolerance test with 75 g of glucose and mixed meals can reduce T levels by up to 20% to 30% (^45^,^46^), confirming these results with mixed meals (^47^). Since our patients did not consume these excessive amounts of glucose and fat, we hypothesise that there was no significant effect on T levels. However, while we await more consistent data, we refer to the recommendations of the international guidelines for a morning fasting test. In any case, several studies have shown that SHBG levels are not affected by glucose or mixed meal intake (^45^,^46^,^47^).

This study has some limitations. Firstly, the cross-sectional design does not allow conclusions regarding the causality of the findings, but it is tempting to speculate that SHBG might be a risk marker based on the associations found. Secondly, the maximum age of the participants included was 67 years, due to the difficulty to identify healthy older subjects without comorbidities, restricting the external validity of our findings to older age groups. Finally, as the subjects were blood donors, we were unable to measure waist circumference as there was no suitable place to do so. In this setting, we were also unable to collect fasting blood glucose and could not use these parameters in other insulin resistance formulas. However, the lack of fasting blood sampling has no effect on SHBG levels, as previously mentioned.

The strengths of the study are the careful exclusion of individuals with comorbidities and obesity from the sample, with the guarantee of a reliable profile of healthy men in our population. Furthermore, we reached an adequate sample size, as previously statistically estimated. Finally, there are no previous studies regarding this issue in our population.

In conclusion, this study showed a substantial age-related increase in SHBG levels, indicating the need for age-specific values. Furthermore, as recommended by guidelines, the development of values for each specific population is highly welcome. Even though all this knowledge is not new, several laboratories including attitude-forming university hospitals do not take this phenomenon into account when reporting male hormone levels. This highlights the need of further calling the attention to this essential subject. Finally, SHBG was inversely correlated with IRI, which suggests complex pathophysiological interrelationships, with possible influences on general health prediction. This needs to be explored in future investigations, including the use of SHBG as a possible biomarker or predictor of chronic metabolic diseases and unfavorable cardiovascular outcomes.

## References

[1] Goldman AL, Bhasin S, Wu FC, Krishna M, Matsumoto AM, Jasuja R (2017). A reappraisal of testosterone's binding in circulation:
physiological and clinical implications. Endocr Rev.

[2] Basaria S (2014). Male hypogonadism. Lancet.

[3] Thaler MA, Seifert-Klauss V, Luppa PB (2015). The biomarker sex hormone-binding globulin - From established
applications to emerging trends in clinical medicine. Best Pract Res Clin Endocrinol Metab.

[4] Kaufman JM, Lapauw B, Mahmoud A, T'Sjoen G, Huhtaniemi IT (2019). Aging and the Male Reproductive System. Endocr Rev.

[5] Wu FC, Tajar A, Pye SR, Silman AJ, Finn JD, O'Neill TW (2008). Hypothalamic-pituitary-testicular axis disruptions in older men
are differentially linked to age and modifiable risk factors: the European
Male Aging Study. J Clin Endocrinol Metab.

[6] Handelsman DJ, Yeap BB, Flicker L, Martin S, Wittert GA, Ly LP (2015). Age-specific population centiles for androgen status in
men. Eur J Endocrinol.

[7] Orwoll E, Lambert LC, Marshall LM, Phipps K, Blank J, Barrett-Connor E (2006). Testosterone and Estradiol among Older Men. J Clin Endocrinol Metab.

[8] Diokno AC (2022). The role of testosterone in men's health: is it time for a new
approach?. Int Urol Nephrol.

[9] Biobanco Covid-19 GPPG/HCPA (2020). Biobanco Covid-19: amostras biológicas.

[10] Bhasin S, Brito JP, Cunningham GR, Hayes FJ, Hodis HN, Matsumoto AM (2018). Testosterone Therapy in Men With Hypogonadism: An Endocrine
Society Clinical Practice Guideline. J Clin Endocrinol Metab.

[11] Whelton PK, Carey RM, Aronow WS, Casey DE, Collins KJ, Dennison Himmelfarb C (2018). 2017 ACC/AHA/AAPA/ABC/ACPM/AGS/APhA/ASH/ASPC/NMA/PCNA guideline
for the prevention, detection, evaluation, and management of high blood
pressure in adults. J Am Coll Cardiol.

[12] Vermeulen A, Verdonck L, Kaufman JM (1999). A critical evaluation of simple methods for the estimation of
free testosterone in serum. J Clin Endocrinol Metab.

[13] Inker LA, Eneanya ND, Coresh J, Tighiouart H, Wang D, Sang Y (2021). New creatinine- and cystatin c-based equations to estimate GFR
without race. N Engl J Med.

[14] Jeppesen J, Hein HO, Suadicani P, Gyntelberg F (2001). Low triglycerides-high high-density lipoprotein cholesterol and
risk of ischemic heart disease. Arch Intern Med.

[15] Salazar MR, Carbajal HA, Espeche WG, Leiva Sisnieguez CE, March CE, Balbín E (2013). Comparison of the abilities of the plasma triglyceride/
high-density lipoprotein cholesterol ratio and the metabolic syndrome to
identify insulin resistance. Diabetes Vasc Dis Res.

[16] Travison TG, Vesper HW, Orwoll E, Wu F, Kaufman JM, Wang Y (2017). Harmonized reference ranges for circulating testosterone levels
in men of four cohort studies in the United States and
Europe. J Clin Endocrinol Metab.

[17] Borges RB, Mancuso AC, Camey SA, Leotti VB, Hirakata VN, Azambuja GS (2021). Power and Sample Size for Health Researchers: uma ferramenta para
cálculo de tamanho amostral e poder do teste voltado a pesquisadores
da área da saúde. Clin Biomed Res.

[18] Krakowsky Y, Conners W, Morgentaler A (2019). Serum concentrations of sex hormone-binding globulin vary widely
in younger and older men: clinical data from a men's health
practice. Eur Urol Focus.

[19] Yeap BB, Marriott RJ, Antonio L, Bhasin S, Dobs AS, Dwivedi G (2021). Sociodemographic, lifestyle and medical influences on serum
testosterone and sex hormone-binding globulin in men from UK
Biobank. Clin Endocrinol (Oxf).

[20] Wan Q, Xie Y, Zhou Y, Shen X (2021). Research progress on the relationship between sex hormone-binding
globulin and male reproductive system diseases. Andrologia.

[21] Handelsman DJ, Sikaris K, Ly LP (2016). Estimating age-specific trends in circulating testosterone and
sex hormone-binding globulin in males and females across the
lifespan. Ann Clin Biochem Int J Lab Med.

[22] Renck AC, Trarbach EB, Brasil S, Frade Costa EM (2024). Sex hormone-binding globulin as more than a biomarker of
metabolic diseases and reproductive disorders: what physicians should
know?. J Biomed Res Environ Sci.

[23] Wang Y (2021). Definition, prevalence, and risk factors of low sex
hormone-binding globulin in US adults. J Clin Endocrinol Metab.

[24] Cooper RS, Kaufman JS, Ward R (2003). Race and genomics. N Engl J Med.

[25] Escott GM, Zingano CP, Ferlin E, Garroni M, Thomé FS, Veronese FJ (2024). Is race adjustment necessary to estimate glomerular filtration
rate in South Brazilians?. J Nephrol.

[26] Feldman HA, Longcope C, Derby CA, Johannes CB, Araujo AB, Coviello AD (2002). Age trends in the level of serum testosterone and other hormones
in middle-aged men: longitudinal results from the massachusetts male aging
study. J Clin Endocrinol Metab.

[27] Mezzullo M, Di Dalmazi G, Fazzini A, Baccini M, Repaci A, Gambineri A (2020). Impact of age, body weight and metabolic risk factors on steroid
reference intervals in men. Eur J Endocrinol.

[28] Kalme T, Koistinen H, Loukovaara M, Koistinen R, Leinonen P (2003). Comparative studies on the regulation of insulin-like growth
factor-binding protein-1 (IGFBP-1) and sex hormone-binding globulin (SHBG)
production by insulin and insulin-like growth factors in human hepatoma
cells. J Steroid Biochem Mol Biol.

[29] Vanbillemont G, Lapauw B, De Naeyer H, Roef G, Kaufman JM, Taes YEC (2012). Sex hormone-binding globulin at the crossroad of body
composition, somatotropic axis and insulin/glucose homeostasis in young
healthy men. Clin Endocrinol (Oxf).

[30] Watts EL, Fensom GK, Smith Byrne K, Perez-Cornago A, Allen NE, Knuppel A (2021). Circulating insulin-like growth factor-I, total and free
testosterone concentrations and prostate cancer risk in 200 000 men in UK
Biobank. Int J Cancer.

[31] Xie L, Wang W (2013). Weight control and cancer preventive mechanisms: Role of insulin
growth factor-1-mediated signaling pathways. Exp Biol Med.

[32] Olivas A, Price RS (2021). Obesity, Inflammation, and Advanced Prostate
Cancer. Nutr Cancer.

[33] Cnop M, Havel PJ, Utzschneider KM, Carr DB, Sinha MK, Boyko EJ (2003). Relationship of adiponectin to body fat distribution, insulin
sensitivity and plasma lipoproteins: evidence for independent roles of age
and sex. Diabetologia.

[34] Simó R, Saez-Lopez C, Lecube A, Hernandez C, Fort JM, Selva DM (2014). Adiponectin upregulates SHBG production: molecular mechanisms and
potential implications. Endocrinology.

[35] Li J, Zheng L, Chan KHK, Zou X, Zhang J, Liu J (2023). Sex hormone-binding globulin and risk of coronary heart disease
in men and women. Clin Chem.

[36] Dhindsa S, Ghanim H, Batra M, Dandona P (2018). Hypogonadotropic hypogonadism in men with
diabesity. Diabetes Care.

[37] Bhasin S, Jasjua GK, Pencina M, D'Agostino R, Coviello AD, Vasan RS (2011). Sex hormone-binding globulin, but not testosterone, is associated
prospectively and independently with incident metabolic syndrome in
men. Diabetes Care.

[38] Ding EL, Song Y, Manson JE, Hunter DJ, Lee CC, Rifai N (2009). Sex hormone-binding globulin and risk of type 2 diabetes in women
and men. N Engl J Med.

[39] Mohammed M, AL-Habori M, Abdullateef A, Saif-Ali R (2018). Impact of metabolic syndrome factors on testosterone and SHBG in
type 2 diabetes mellitus and metabolic syndrome. J Diabetes Res.

[40] Souteiro P, Belo S, Oliveira SC, Neves JS, Magalhães D, Pedro J (2018). Insulin resistance and sex hormone-binding globulin are
independently correlated with low free testosterone levels in obese
males. Andrologia.

[41] Pataky MW, Young WF, Nair KS (2021). Hormonal and metabolic changes of aging and the influence of
lifestyle modifications. Mayo Clin Proc.

[42] Wu Z, Cui H, Li W, Zhang Y, Liu L, Liu Z (2022). Comparison of three non-insulin-based insulin resistance indexes
in predicting the presence and severity of coronary artery
disease. Front Cardiovasc Med.

[43] Che B, Zhong C, Zhang R, Pu L, Zhao T, Zhang Y (2023). Triglyceride-glucose index and triglyceride to high-density
lipoprotein cholesterol ratio as potential cardiovascular disease risk
factors: an analysis of UK biobank data. Cardiovasc Diabetol.

[44] Ozarda Y (2016). Reference intervals: current status, recent developments and
future considerations. Biochem Med (Zagreb).

[45] Gagliano-Jucá T, Li Z, Pencina KM, Beleva YM, Carlson OD, Egan JM (2019). Oral glucose load and mixed meal feeding lowers testosterone
levels in healthy eugonadal men. Endocrine.

[46] Meikle AW, Stringham JD, Woodward MG, McMurry MP (1990). Effects of a fat-containing meal on sex hormones in
men. Metabolism.

[47] Lehtihet M, Arver S, Bartuseviciene I, Pousette A (2012). S-testosterone decrease after a mixed meal in healthy men
independent of SHBG and gonadotrophin levels. Andrologia.

